# COMMENT: HOSPITALIZATIONS FOR CHOLECYSTITIS AND CHOLELITHIASIS IN THE STATE OF RIO GRANDE DO SUL, BRAZIL

**DOI:** 10.1590/0102-6720201600040022

**Published:** 2016

**Authors:** José CABALLERO, Miguel TRESIERRA, Melissa DIAZ

**Affiliations:** Universidad Privada Antenor Orrego, Trujillo, Perú.

COMMENTS

Dear Editor:

We have read with interest the recently research published by Nunes EC et al^1^ and we believe that this is a very interesting study considering how prevalent is gallbladder calculus disease in Latin America. However, we would like to make some comments, as there are some aspects that can be discussed.

1. Can cholecystitis and cholelithiasis be integrated into the same universe? We believe not. The first is an emergency condition and requires, according to its state at the time of admission, previous medical treatment and surgery, which can be postponed and re-enter in some other time; the second is an elective admission. Cholecystitis will be more likely to have more hospitalizations than cholelithiasis, surgical solution decreases the number of hospitalizations.
2. The authors refer to, as one of the variables of the study, the total number of hospitalizations, but it is not well understood why the "hospitalization/general population" indicator, as the proportional difference of this relative frequency is the same as the absolute number. It is more convenient to measure the specific rate of hospitalizations by age group that makes the economic analysis of the in-hospital stay more realistic. It is also useful to measure the concentration of hospitalizations by case and adjust it by variables that may be confusing such as socioeconomic status, educational level, purchasing power, nutrition education, adherence to treatment; the latter is at the same time influenced by work schedule, food culture, and the ability to decide the home meal. These variables that could be intervening are not being analyzed in the study: the number of times of in-patient admission, and the hospital stay. For a more updated economic analysis, medical expenses are no longer made on the basis of an event but on the basis of a case, therefore it is necessary to differentiate hospitalizations per incident case to not confuse hospitalizations of prevalent cases. This improves the economic analysis and allows to propose interventions on the issue of costs of hospitalization for cholecystitis and cholelithiasis.
3. Regarding the severity of gallbladder calculus disease, the authors hypothesize that gravity would be associated with the anthropometric characteristics, distribution of body fat and pain threshold; however, on this point there are other variables that may explain the severity conditions in patients coming to the emergency services and these are due to other variables such as self-medication, access to health services, patients' idiosyncrasies, among others; which often involve a delay in attending a hospital with a consequent increase in the severity of cholecystitis.
4. In terms of mortality, this was elevated in the elderly; probably associated with comorbidity, greater severity of the disease at hospital admission and less physiologic reserve, feature of this patient group; on the other hand there wasn't any exclusion criteria, which means there were mixed diagnoses such as cholangitis, gallbladder cancer, acute pancreatitis, among others. Additionally, early cholecystectomy in these patients could result in morbidity up to 41% and perioperative mortality up to 18%^2^.
5. Considering the surgical average time of the open and laparoscopic cholecystectomy, it is claimed to be similar between the elderly and the young; however, we must differentiate between an elective or an emergency surgery for these groups of people; it is known that the elderly have an increased presence of risk factors for conversion of laparoscopic cholecystectomy to open surgery as fibrosis of the gallbladder wall due to repetitive cholecystitis which likewise causes a retracted gallbladder, increased likelihood of adherence syndrome by the history of previous surgeries^3^.
6. There was restriction on the study to not established if the surgery was performed on the context of an elective or emergency surgery, the study refers or distinguishes cholecystitis and cholelithiasis, so that could indirectly approach more accurate data distribution, which means that the patients with cholelithiasis are candidates for elective surgery and patients with acute conditions, meaning cholecystitis go to emergency surgery; an analysis from this point of view would have been more enlightening and not analyze it as a whole. The study doesn't classify patients according its severity, i.e. Acute Cholecystitis grade I, II or III according to Tokyo guidelines, therefore we cannot tell whether the treatment was early, late or in between, which may affect mortality.
7. It is referred that there was a major expense on children under 4 years coursed with cholelithiasis or cholecystitis, probably due to the oddness of thinking of cholelithiasis as a diagnosis in this group and it also requires further studies and a more complex treatment than adults^4^.
