# Supplementary material for: COMMENT: HOSPITALIZATIONS FOR CHOLECYSTITIS AND CHOLELITHIASIS IN THE STATE OF RIO GRANDE DO SUL, BRAZIL
Source: Arq Bras Cir Dig. 2016 Nov-Dec;29(4):297–8. doi: 10.1590/0102-6720201600040022 (PMC5225878; doi:10.1590/0102-6720201600040022)
Supplement: Related texts

# REPLY

Dear Editor:

In response to the comments and issues raised for discussion by Caballero, Tresierra and Diaz, respecting the same order of presentation of the items, we have to consider that:

1. Our focus approached hospitalizations for cholecystitis (ICD-10 K80) and cholelithiasis (ICD-10 K81) considering both part of the same universe of gallbladder disease. As the immense majority of cases of cholecystitis is due to obstruction of the bile duct by the appearance of gallstones (cholelithiasis), it seemed appropriate to present together the panorama of hospitalizations for both diseases.
2. We agree that other variables may be incorporated in the analysis. However, our data source was the Hospital Information System of the Brazilian National Health System (SIH/SUS, in Portuguese). This information system was developed in the 1980s based on the payment of all hospital bills of the Brazilian public system. This system is of mandatory use by all service providers in the country and has an average of 11 million hospitalizations per year. The data are administrative, secondary, and are publicly available on the internet.
3. Given their purpose, their unit of analysis is the hospitalization and not the patient. Due to their database structure, it lacks several variables such as the number of times of in-patient admission, socioeconomic status, educational level, purchasing power, nutrition education, adherence to treatment of each patient. We understand that in Table 2 we present specific indicators by age (admissions per 10,000 people/year).
4. In fact, other variables could explain the severity of the condition. At this point, we quote only the reference of Peron, Schliemann and Almeida. Understanding the reasons for the refusal of cholecystectomy in patients with cholelithiasis: how to help them in their decision? ABCD Arq. Bras. Cir. Dig. 2014 Apr-Jun; 27 (2): 114-19.
5. The SIH/SUS does not have data on comorbidities of hospitalized patients. However, considering that they are about 20,000 hospitalizations per year for ICD-10 K80 and K81 for a three-year period, all performed by the Brazilian public system in Rio Grande do Sul, we understand that this number is representative of population profile that we intended to present.
6. This study did not aimed to examine differences between elective or emergency admissions, which could be further realized.
7. The SIH/SUS does not have data such as the classification of patients according to Tokyo guidelines. These data are in the medical records of the patients and not in the secondary database that was built for all kinds of stay and not just cholelithiasis and cholecystitis.
8. We agree, but as mentioned, the SIH/SUS does not have these variables. To obtain such data requires a designed study for the use of primary data. This is not the case of our article, which uses secondary data publicly available on the internet.
9. Finally, we would like to thank the comments made. They contributed to further examination of the matter in question from other perspectives.

Emeline C Nunes, Roger S Rosa and Ronaldo Bordin
